# Post-COVID-19 Vaccine Thromboembolic Complication in the Setting of Newly Diagnosed May-Thurner Syndrome

**DOI:** 10.7759/cureus.55746

**Published:** 2024-03-07

**Authors:** Fadila Noor, Hamza Khan, Maryam Hanoodi, Muhammad Ali, Valerie Cluzet

**Affiliations:** 1 Internal Medicine, Vassar Brothers Medical Center/Nuvance Health, Poughkeepsie, USA; 2 Infectious Diseases, Vassar Brothers Medical Center/Nuvance Health, Poughkeepsie, USA

**Keywords:** vaccine-induced immune thrombotic thrombocytopenia (vitt), may-thurner's syndrome, therapeutic anticoagulation, venous thromboembolism (vte), covid-19 vaccine complication

## Abstract

May-Thurner syndrome (MTS) can lead to deep venous thrombosis (DVT) in the left lower extremity, and it is often triggered by factors such as surgery or pregnancy. We present a rare case where the risk factor for thromboembolism in MTS is a complication from COVID-19 vaccination. A 44-year-old female who presented with fatigue, fever, and myalgia had developed thromboembolism as a complication of the Johnson & Johnson COVID-19 vaccine. Diagnostic criteria for vaccine-induced immune thrombotic thrombocytopenia (VITT) should be considered in such cases that include symptoms within 5-30 days post vaccination, elevated D-dimer, and thrombosis. Treatment involved anticoagulants and intervention for MTS included thrombectomy and stent placement. Recognition of post-COVID-19 vaccination complications such as VITT is crucial for early intervention and patient awareness during vaccination decisions.

## Introduction

May-Thurner syndrome (MTS) is the compression of the left common iliac vein by the right common iliac artery against the fifth lumbar vertebra. The chronic pulsatile action of the artery irritates the endothelium of the vein, eventually leading to venous spur formation and, consequently, clot formation. MTS predisposes to extensive deep venous thrombosis (DVT) of the left lower extremity [1,2]. It is estimated to be the cause of about 2%-3% of all DVTs. The incidence appears to be higher in women than in men. Additionally, patients are usually asymptomatic unless provoked by a predisposing condition for hypercoagulability, such as surgery, pregnancy, or postpartum state [1]. Here, we discuss a rare case where the risk factor for thromboembolism in MTS is observed as a complication from COVID-19 vaccination.

## Case presentation

A 44-year-old female with a history of endometriosis presented to the hospital with complaints of fatigue, fever, headache, and myalgias for five days. She had received the Johnson & Johnson COVID-19 vaccine six days before presentation. The day after the vaccination, she experienced swelling and pain at the site of injection associated with numbness of the fingertips of the left hand. This was followed by fatigue, fever (time to peak drug concentration, Tmax: 103oF), chills, and myalgias. Five days later, she noticed purple discoloration of her left lower extremity, swelling, difficulty walking, and pain. With elevation of her left lower extremity, she experienced some pain relief and improvement of swelling and discoloration. She had no reactions to vaccinations in the past, recent travel, personal or family history of hypercoagulability, recent surgery, oral contraceptive use, or tobacco use.

**Figure 1:**
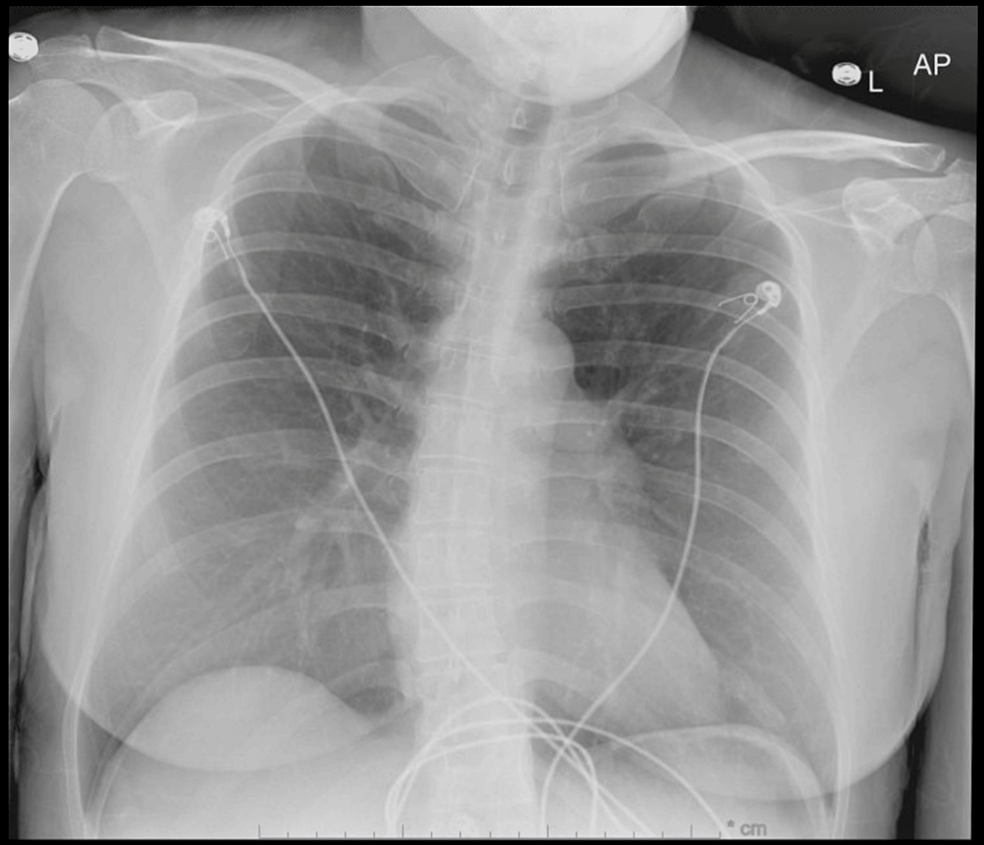
An X-ray with an anteroposterior view of the chest showing hyperaerated lungs

**Figure 2:**
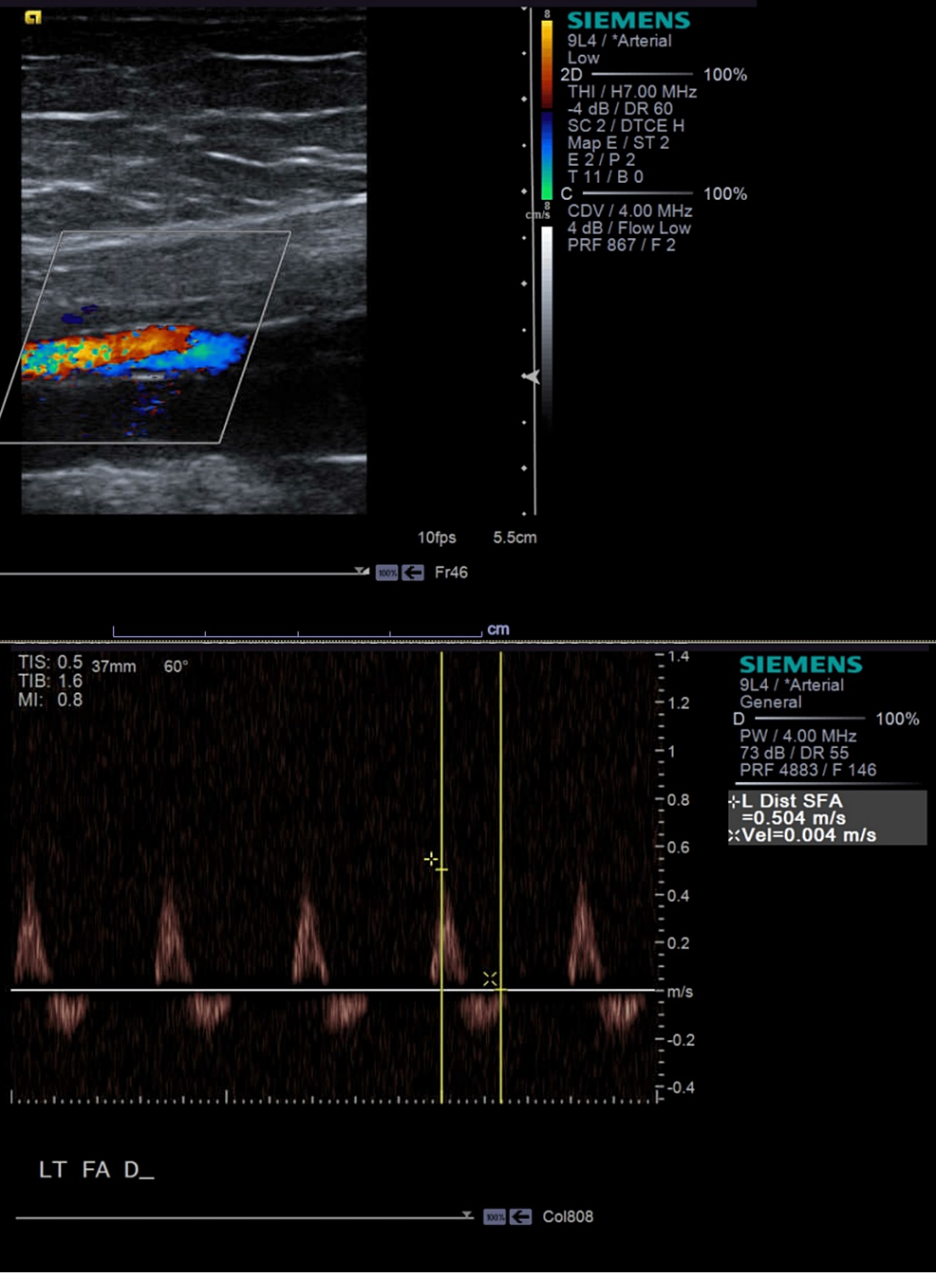
The left lower extremity ultrasound duplex, spectral Doppler analysis, and color flow Doppler revealing stenosis in the distal superficial femoral artery

**Figure 3:**
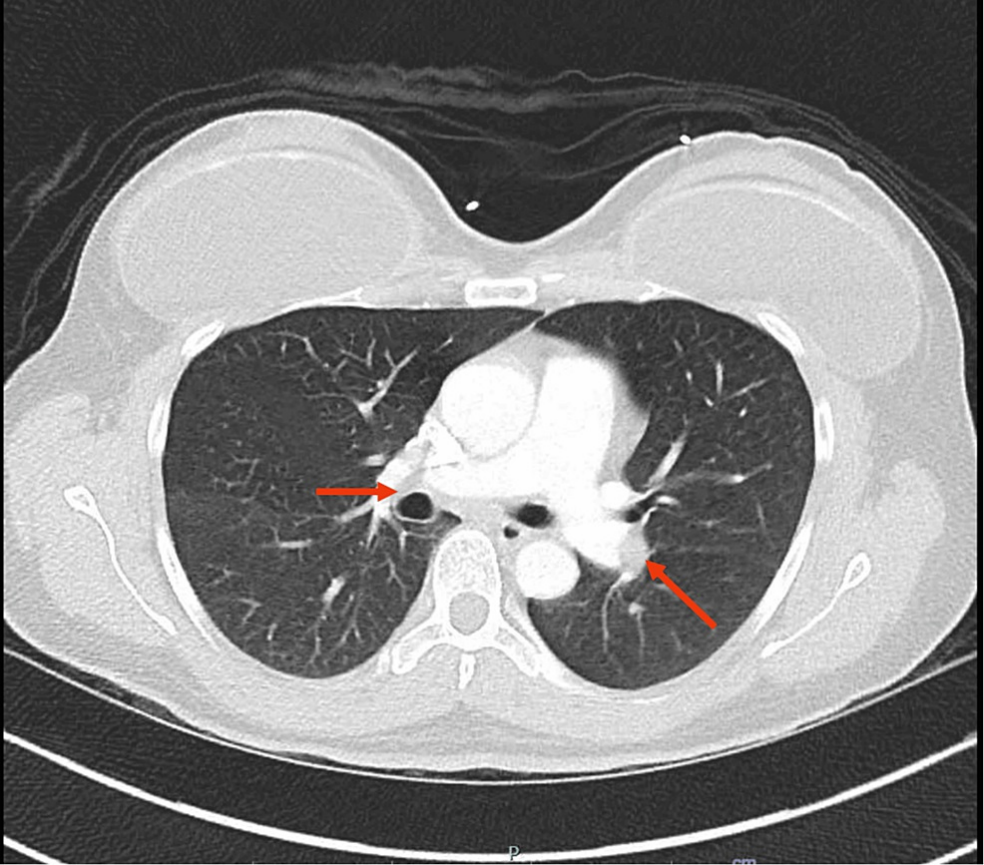
A CT angiogram chest with red arrows revealing filling defects suggestive of bilateral pulmonary emboli

**Figure 4:**
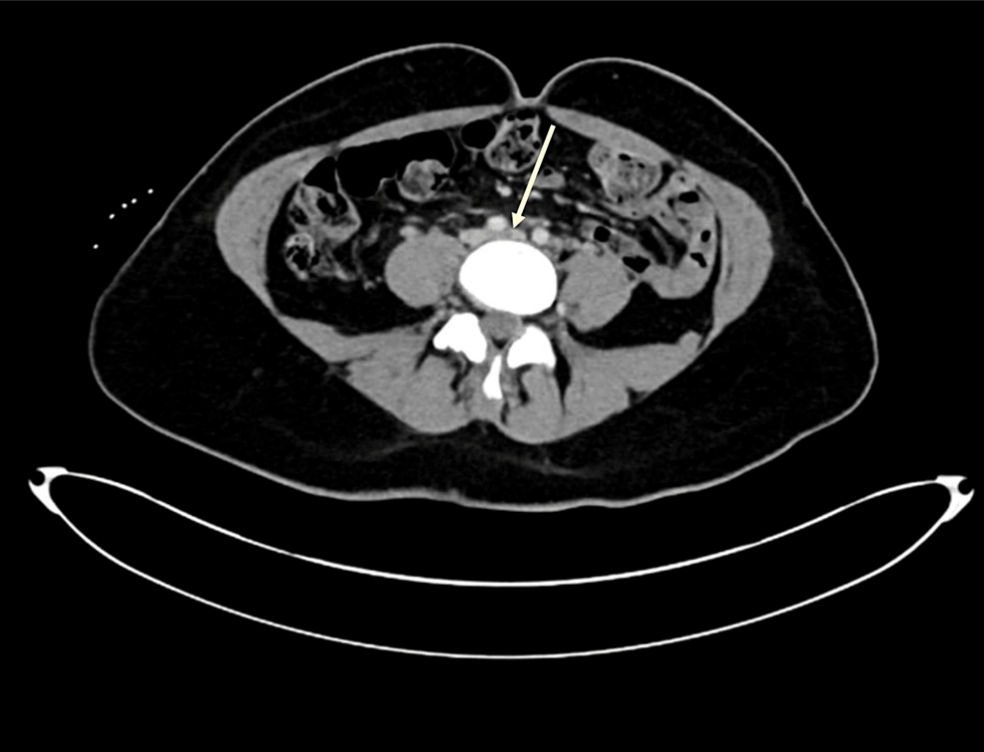
A CT abdomen and pelvis venogram with an arrow showing compression of the left common iliac vein from the right common iliac artery

On presentation, the patient was afebrile, with blood pressure of 136/77, pulse of 120/min, respiratory rate of 18/min, and saturating well (97%) on room air. The physical exam revealed mottling of the left lower extremity and slight mottling of the left forearm. Her radial, femoral, and pedal pulses were palpable bilaterally. She had a left lower extremity pitting edema up to the hip and tenderness to palpation up to the left thigh. Initial lab work exhibited hemoglobin of 12.7 g/dL, platelet count of 201 x109/L, and an elevated D-dimer 3571 ng/mL D-dimer units. Initial EKG showed sinus tachycardia at 128 bpm. A chest X-ray presented hyperaerated lungs without acute infiltrate (Figure 1). Ultrasound (US) Doppler of the left lower extremity showed no evidence of DVT. US arterial Doppler showed stenosis in the distal left superficial femoral artery (Figure 2). A CT angiogram chest revealed bilateral pulmonary emboli (PE) (Figure 3). She was started on therapeutic enoxaparin and transferred to our hospital for further evaluation by vascular surgery and hematology. An echocardiogram showed a normal ejection fraction without evidence of a right heart strain. As the patient began experiencing a headache, she underwent magnetic resonance venography (MRV) head, which helped rule out cavernous sinus thrombosis. To assess for iliofemoral DVT, the patient underwent CT abdomen and pelvis venogram, which revealed compression of the left common iliac vein from the right common Iliac artery (Figure 4); acute thrombus in the left common iliac vein extending into the inferiormost vena cava; extensive thrombus seen in the left external iliac vein, left common femoral vein, great saphenous vein, and left superficial femoral vein. Anticoagulation was subsequently changed to intravenous heparin infusion. Vascular surgery proceeded with mechanical thrombectomy of the left iliofemoral segment and left iliac vein stenting, which resulted in the improvement of left lower extremity pain and swelling. Thrombophilia work-up with factor V, antinuclear antibodies (ANA), antiphospholipid syndrome panel, and homocysteine levels were within normal limits. On postoperative day two, the patient was transitioned to an apixaban loading dose. As the patient remained hemodynamically stable, she was recommended to use a left lower extremity ACE compression bandage (3M Company, Saint Paul, MN) for edema, which overall had improved post procedure. She was discharged with a close vascular surgery and hematology follow-up.

## Discussion

MTS confers a predisposition for extensive DVT of the left lower extremity [1,2]. The compression of the left iliac vein leads to venous thrombosis secondary to impaired venous return owing to stasis. Concurrently, compression-induced endothelial injury gives rise to intimal hyperplasia. Both of these processes are components of Virchow's triad [2]. The gold standard for diagnosis is usually CT, MRI, or conventional venography to detect the location of iliac vein thrombosis and compression [2].

COVID-19 vaccination can lead to a rare adverse effect of vaccine-induced thrombosis. Vaccine-induced immune thrombotic thrombocytopenia (VITT) with venous thrombosis is one of the rare complications associated with adenoviral vector-based COVID-19 vaccinations [3]. Adenoviral vaccines are preferred in resource-limited countries because of a lack of cold storage requirements and ease of vaccination with a single dose schedule. Therefore, when comparing the risks and benefits of COVID-19 vaccination, it has been deemed to be overall beneficial [4]. There are reports from multiple countries revealing rare thromboembolic incidences, especially cavernous sinus venous thrombosis (CSVT) or DVT of the lower limbs post adenoviral vector vaccination for COVID-19 [5]. It is hypothesized that platelet-activating antibodies with vaccination involve a strong inflammatory stimulation. There may be a possible interaction between anionic-free DNA and the recombinant adenoviral vaccine with cationic platelet factor 4 (PF4). This forms complexes exposing an epitope to which anti-PF4/heparin antibodies bind to elicit an immunological response similar to heparin-induced thrombocytopenia (HIT) with thrombosis and thrombocytopenia [3,6].

There are diagnostic criteria for the likelihood of VITT, which include 1) symptoms within five to 30 days after vaccination; 2) presence of thrombosis; 3) D-dimer level > 4000 fibrinogen equivalent units; 4) thrombocytopenia; and 5) positive anti-PF4 HIT antibodies observed on enzyme-linked immunosorbent assay (ELISA) [3]. In our case, the patient did not have thrombocytopenia, but she did have thrombosis with pulmonary embolus and extensive iliofemoral thrombosis. Unfortunately, her anti-PF4 levels were not checked, which may have been helpful in ascertaining that this was a complication of vaccination. Treatment for VITT involves nonheparin anticoagulants, intravenous immunoglobulin, and potentially plasma exchange to reduce antibody load [3,5]. In cases where thrombocytopenia is present, it is recommended to avoid platelet transfusions as the transfused platelets would become a substrate for coagulopathy with antibody-mediated platelet activation [5]. The treatment for MTS initially requires medical management with anticoagulation, analgesia, and limb elevation. This is followed by interventions such as catheter-directed thrombolysis, percutaneous or surgical thrombectomy, or stent placement with the goal of reducing thrombus burden and preventing propagation [5].

## Conclusions

It is essential to recognize post-COVID-19 vaccination complications such as VITT. Early recognition and intervention are important in reducing morbidity and mortality. Although COVID-19 vaccines are generally considered safe and effective, it is crucial to involve patients in shared decision-making regarding vaccination, discussing the possibility of experiencing thrombotic events, especially when considering adenoviral vector vaccines.
